# The Diagnostic and Prognostic Role of Biomarkers in Mild Traumatic Brain Injury: An Umbrella Meta-Analysis

**DOI:** 10.3390/brainsci15060581

**Published:** 2025-05-28

**Authors:** Ioannis Mavroudis, Foivos Petridis, Dimitrios Kazis, Alin Ciobica, Gabriel Dăscălescu, Antoneta Dacia Petroaie, Irina Dobrin, Otilia Novac, Ioana Vata, Bogdan Novac

**Affiliations:** 1 Department of Neuroscience, Leeds Teaching Hospitals, NHS Trust, Leeds LS9 7TF, UK; i.mavroudis@nhs.net; 2 Third Department of Neurology, Aristotle University of Thessaloniki, 541 24 Thessaloniki, Greece; f_petridis83@yahoo.gr (F.P.); dimitrios.kazis@gmail.com (D.K.); 3 Faculty of Biology, “Alexandru Ioan Cuza” University of Iași, 700506 Iași, Romania; gabidascalescu2001@gmail.com; 4 “Ioan Hăulică” Institute, Apollonia University of Iași, 700511 Iași, Romania; 5 CENEMED Platform for Interdisciplinary Research, “Grigore T. Popa” University of Medicine and Pharmacy of Iasi, 700115 Iași, Romania; 6 Academy of Romanian Scientists, Iasi Branch, 700481 Iași, Romania; 7 Faculty of Medicine, University of Medicine and Pharmacy “Grigore T. Popa”, 700115 Iasi, Romania; pantoneta@yahoo.com (A.D.P.); irinadobrin2002@gmail.com (I.D.); otilia76@gmail.com (O.N.); bogdannvc@gmail.com (B.N.); 8 Faculty of Dental Medicine, Apollonia University, Pacurari Street 11, 700511 Iași, Romania

**Keywords:** mild traumatic brain injury, concussion, biomarkers, S100B, GFAP, UCH-L1, neurofilament light chain, Tau protein, meta-analysis, diagnostic accuracy, traumatic brain injury detection

## Abstract

Background: Mild traumatic brain injury (mTBI), commonly known as concussion, is a major public health issue characterized by subtle neuronal damage that traditional imaging techniques such as computed tomography (CT) and magnetic resonance imaging (MRI) often fail to detect. Fluid biomarkers have emerged as promising diagnostic and prognostic tools for mTBI. Objectives: This umbrella meta-analysis aims to evaluate the diagnostic accuracy and clinical utility of the key fluid biomarkers, S100B, glial fibrillary acidic protein (GFAP), ubiquitin carboxy-terminal hydrolase L1 (UCH-L1, neurofilament light chain (NfL)) and tau protein, in detecting mTBI and to clarify their roles as screening, confirmatory, and complementary indicators. Methods: A systematic review was performed using PubMed, Web of Science, Scopus, and Cochrane to identify the published meta-analyses that assessed the biomarkers in mTBI. Sensitivity, specificity, and diagnostic odds ratios were then calculated using random-effects models. Heterogeneity was evaluated with the I^2^ statistic, and publication bias was assessed via funnel plots. The results of S100B demonstrated high sensitivity (91.6%) but low specificity (42.4%), making it an effective rule-out biomarker to minimize unnecessary CT scans. In contrast, GFAP exhibited moderate sensitivity (84.5%) with improved specificity (61.0%), supporting its role in confirming mTBI diagnoses. UCH-L1 revealed a sensitivity of 86.7% alongside low specificity (37.3%), indicating its potential as a complementary marker. Additionally, the NfL levels were notably elevated in sports-related concussions, while the diagnostic utility of tau protein remains inconclusive due to limited available data. Conclusions: The findings underscore the clinical promise of fluid biomarkers in the management of mTBI. S100B and GFAP are particularly valuable as screening and confirmatory markers, respectively. Nonetheless, further standardization of biomarker thresholds and additional longitudinal studies are necessary to validate the roles of UCH-L1, NfL, and Tau protein. The integration of these biomarkers into a multimodal diagnostic panel may enhance mTBI detection accuracy and facilitate improved patient stratification and management.

## 1. Introduction

Mild traumatic brain injury (mTBI), commonly referred to as concussion, is a prevalent neurological condition characterized by a transient disruption of brain function due to external mechanical forces. Despite being labeled as “mild”, mTBI can lead to a spectrum of clinical manifestations, ranging from brief alterations in consciousness to persistent cognitive, emotional, and physical symptoms [1]. Understanding its epidemiology, pathophysiology, diagnostic challenges, and management strategies is crucial for addressing the public health implications associated with mTBI.

Globally, mTBI represents a significant public health concern. In the United States alone, approximately 214,000 TBI-related hospitalizations occurred in 2020, highlighting its widespread impact [2]. The variability in reported incidence rates across different regions can be attributed to differences in diagnostic criteria, reporting practices, and healthcare access. Estimates suggest that the annual incidence of mTBI ranges from 200 to 300 per 100,000 persons per year for hospitalized cases, with substantially higher rates when non-hospitalized cases are included [3].

Demographically, mTBI exhibits a higher prevalence among males and younger age groups, particularly adolescents and young adults, often due to increased participation in contact sports, occupational hazards, and risk-taking behaviors [4]. Falls, motor vehicle accidents, sports-related injuries, and assaults are among the most common causes of mTBI [5]. Although frequently associated with athletes and military personnel, mTBI affects individuals across all age groups and socioeconomic backgrounds.

The pathophysiological mechanisms underlying mTBI involve complex biomechanical processes that result in neuronal and glial perturbations. The rapid acceleration/deceleration forces exerted on the brain during injury can lead to diffuse axonal injury, disruption of neuronal membranes, and alterations in cerebral blood flow [6]. These changes contribute to the clinical manifestations of mTBI, which include headaches, dizziness, cognitive impairments (such as memory and attention deficits), emotional disturbances (including irritability and depression), and sleep disturbances [7]. While many individuals experience symptom resolution within days to weeks, a subset may develop persistent post-concussive symptoms, leading to prolonged functional impairments [8].

Diagnosing mTBI presents significant challenges due to the often subtle and subjective nature of the symptoms, coupled with a lack of definitive diagnostic tools. Traditional neuroimaging modalities, such as computed tomography (CT) scans and magnetic resonance imaging (MRI), frequently fail to detect abnormalities in mTBI cases, as these techniques are primarily designed to identify macroscopic structural damage [9]. This has led to a growing interest in fluid biomarkers, which can serve as objective measures of brain injury.

Several candidate biomarkers have emerged, including S100B, glial fibrillary acidic protein (GFAP), ubiquitin carboxyl-terminal hydrolase L1 (UCH-L1), neurofilament light chain (NfL), and tau protein being selected according to the following criteria: have been evaluated in at least two published meta-analyses or systematic reviews; provide specific measurements of neuronal, astrocytic, or axonal injury; and have demonstrated clinical relevance through validation in an emergency setting (e.g., FDA or CE approval for rapid diagnostic tests).

S100B is a calcium-binding protein predominantly found in astrocytes. S100B has been extensively studied as a potential biomarker for mTBI. Elevated serum levels of S100B have been associated with blood–brain barrier disruption and astrocytic activation following brain injury [10]. However, its specificity is limited due to extracerebral sources, such as adipose tissue and muscle, which can also release S100B [11].

GFAP is an intermediate filament protein that is specific to astrocytes. Following mTBI, elevated levels of GFAP have been detected in both serum and cerebrospinal fluid, reflecting astroglial injury [12]. Studies suggest that GFAP levels correlate with injury severity and may aid in distinguishing mTBI from other conditions [13].

UCH-L1 is a neuronal protein involved in protein degradation pathways. Increased serum concentrations of UCH-L1 have been observed following mTBI, suggesting its potential as a biomarker for neuronal injury. Its rapid elevation post-injury and association with injury severity highlight its diagnostic utility [14].

NfL is a structural protein component of axons, and its presence in biofluids indicates axonal damage. Elevated serum and cerebrospinal fluid levels of NfL have been linked to mTBI, with studies suggesting its potential in assessing injury severity and predicting long-term outcomes [15].

Tau is a microtubule-associated protein that is implicated in maintaining neuronal cytoskeleton integrity. Following mTBI, tau protein levels can become elevated, reflecting neuronal damage. Studies are investigating its role as a biomarker, particularly in its correlation with long-term neurodegenerative changes [16].

Despite the promise of these biomarkers, several unresolved issues remain, including the variability in biomarker kinetics, the influence of extracerebral sources, and the standardization of assay methodologies [17]. Further research is needed to validate these biomarkers and integrate them into clinical practice effectively.

The management of mTBI primarily involves symptomatic treatment and patient education. Initial management focuses on physical and cognitive rest, followed by a gradual return to normal activities as symptoms permit [18]. Multidisciplinary approaches, including physical therapy, cognitive rehabilitation, and psychological support, may be necessary for individuals with persistent symptoms [19].

The prognosis following mTBI is generally favorable, with most individuals experiencing symptom resolution within weeks. However, a subset of patients may develop persistent post-concussive symptoms, leading to chronic impairments. Factors influencing prognosis include the severity of the initial injury, prior history of mTBI, pre-existing neurological or psychiatric conditions, and genetic predispositions [20].

Given the expanding body of research on mTBI biomarkers, synthesizing the available evidence is essential for guiding clinical decision-making. Traditional meta-analyses have evaluated individual biomarkers, yet variability in study methodologies, sample populations, and biomarker thresholds complicate direct comparisons [13]. An umbrella meta-analysis, which integrates data from multiple meta-analyses and systematic reviews, offers a robust approach to evaluating the overall diagnostic accuracy and clinical utility of biomarkers in mTBI.

This study aims to conduct an umbrella meta-analysis that assesses the diagnostic performance of key biomarkers, including S100B, GFAP, UCH-L1, NfL, and tau protein, in detecting mTBI. By consolidating the findings from existing meta-analyses, we seek to provide a comprehensive evaluation of their sensitivity, specificity, and clinical applicability. Furthermore, we aim to identify potential sources of heterogeneity, conduct subgroup analyses, and assess the overall quality of evidence.

## 2. Materials and Methods

To frame our research question, we applied an adapted PICO structure: we included adult and pediatric patients with suspected mTBI, defined by a Glasgow Coma Scale (GCS) score of 13–15, as our population (P); we evaluated the measurement of serum biomarkers of neuronal, astrocytic and axonal injury (specifically S100B, GFAP, UCH-L1, NfL, and tau protein) as our intervention (I); we compared these results against cranial CT findings or, alternatively, against a benchmark biomarker such as S100B (C); and we focused on key diagnostic performance outcomes (sensitivity, specificity, and the diagnostic odds ratio (DOR)) as our endpoints (O).

### 2.1. Study Selection and Data Sources

To evaluate the diagnostic accuracy of blood-based biomarkers for mild traumatic brain injury (mTBI), we conducted an umbrella meta-analysis of the existing systematic reviews and meta-analyses. A comprehensive search was performed through the PubMed, Web of Science, Scopus, and Cochrane databases to identify relevant studies published up to March 2025. Our primary focus was on five biomarkers—glial fibrillary acidic protein (GFAP), ubiquitin C-terminal hydrolase-L1 (UCH-L1), S100 calcium-binding protein B (S100B), tau, and neurofilament light chain (NfL)—and their ability to detect intracranial abnormalities on CT scans in patients with mild TBI. Only systematic reviews containing quantitative synthesis (meta-analysis) were included. For reviews reporting only descriptive data, we extracted only the qualitative conclusions without including them in the statistical pool. Data were extracted independently by two authors (I.M. and F.P.) using a standardized form. Discrepancies were resolved by discussion, and if consensus was not reached, a third author (D.K.) made the final decision.

### 2.2. Inclusion and Exclusion Criteria

Studies were included if they were systematic reviews or meta-analyses that specifically assessed the diagnostic accuracy of one or more of the selected biomarkers in mild TBI patients. Eligible studies needed to report sensitivity, specificity, and confidence intervals (CIs) and include data from at least two independent studies per biomarker.

We excluded studies that focused on moderate to severe TBI, as well as those lacking quantitative diagnostic accuracy data. Case reports, narrative reviews, and studies that did not meet the systematic review standards were also excluded.

### 2.3. Data Extraction and Statistical Analysis

For each eligible study, we extracted the key details, including the study’s authors, publication year, biomarker cutoff values, and diagnostic accuracy measures. Sensitivity and specificity estimates were recorded alongside their respective 95% confidence intervals. When available, we also collected subgroup data, such as the differences in biomarker performance based on the patient’s age, clinical setting, and time from injury to biomarker measurement.

A random-effects meta-analysis was performed using the restricted maximum likelihood (REML) method to account for between-study variability. Forest plots were generated to visualize the pooled sensitivity and specificity of each biomarker. To assess heterogeneity, we calculated the I^2^ statistic, where values of 0–40% indicated low heterogeneity, 41–75% suggested moderate heterogeneity, and values above 75% indicated high heterogeneity. In addition, we computed the diagnostic odds ratio (DOR), a measure of how well each biomarker differentiates between patients with or without intracranial pathology, using the formula:

DOR = Sensitivity × (1 − Specificity)(1 − Sensitivity) × SpecificityDOR = (1 − Sensitivity) × SpecificitySensitivity × (1 − Specificity)

We translated the pooled cutoff values into a simple three-tier scoring system: low, moderate, and high risk. For each biomarker, concentrations below the first threshold were scored 0, those between the first and second threshold scored 1, and those above the second threshold scored 2. Summing these five scores yields a total CDSS score from 1 to 10. In practice, scores 0–2 indicate a low probability (outpatient observation), 3–5 moderate probabilities (consider neuroimaging), and 6–10 high probabilities (recommend urgent imaging and possible admission) of intracranial injury.

To further explore the overall diagnostic performance of each biomarker, we generated summary receiver operating characteristic (SROC) curves. These curves provide a graphical representation of sensitivity versus 1—specificity, offering insight into the trade-off between true positive and false positive rates.

### 2.4. Subgroup and Sensitivity Analyses

Recognizing that biomarker performance can vary across different patient populations and clinical contexts, we conducted several subgroup analyses. These included the following:

- Comparisons between high and low biomarker cutoff values;
- Differences between pediatric and adult populations;
- Impact of time from injury to biomarker measurement.

A leave-one-out sensitivity analysis was also performed to test the robustness of our findings. This involved systematically removing one study at a time and recalculating the pooled estimates to determine whether any single study disproportionately influenced the results.

### 2.5. Risk of Bias and Quality Assessment

The quality of the included meta-analyses was assessed using the AMSTAR-2 (A Measurement Tool to Assess Systematic Reviews) framework, which evaluates the methodological rigor of systematic reviews. Individual studies within each meta-analysis were further assessed using the QUADAS-2 tool, which examines the potential sources of bias related to patient selection, as well as the validity of the index test, reliability of the reference standard, and the timing of patient inclusion.

### 2.6. Final Study Selection

Our initial search identified 52 potential studies, of which 23 met the inclusion criteria. GFAP, UCH-L1, and S100B had sufficient data for meta-analysis, allowing us to calculate the pooled diagnostic accuracy estimates. However, tau and NfL had only one eligible meta-analysis each, limiting our ability to perform an umbrella meta-analysis for these biomarkers. Instead, for tau and NfL, we summarized the findings from their respective meta-analyses, highlighting their reported diagnostic performance.

By synthesizing data from multiple meta-analyses, this study aimed to provide a comprehensive and high-level evaluation of the role of blood biomarkers in mild TBI diagnosis, offering valuable insights for clinical decision-making.

## 3. Results

### 3.1. Selection Process (PRISMA Guidelines)

**Figure 1:**
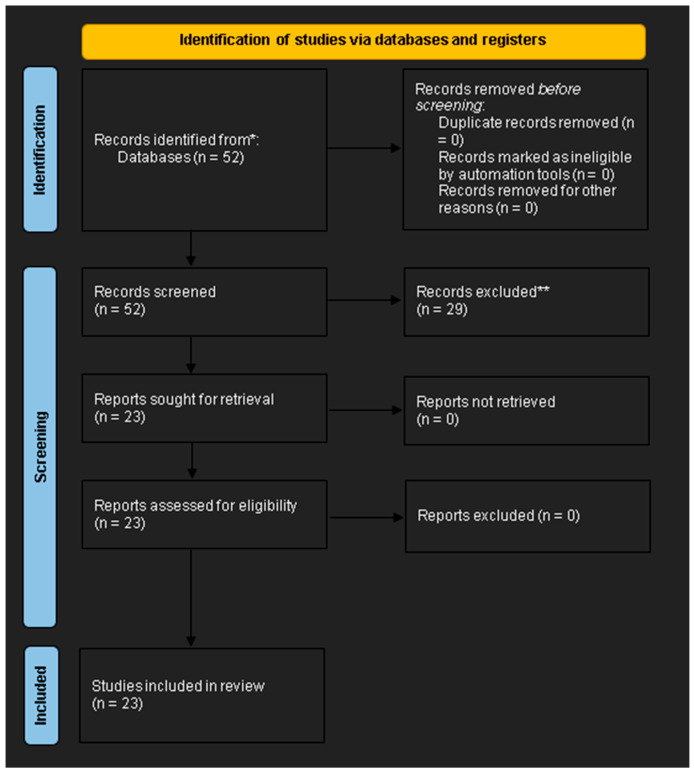
PRISMA flowchart (designed according to the guidelines from [21] and the PRISMA 2020 statement website).

The literature search and selection were performed in accordance with the PRISMA (Preferred Reporting Items for Systematic Reviews and Meta-Analyses) recommendations [21], with a corresponding flowchart shown in Figure 1. This review was not registered in PROSPERO, as data extraction and analysis were completed prior to the decision to register the protocol. What follows is a description of the four key phases of the systematic review: identification, screening, eligibility assessment, and final inclusion of studies.

The database searches retrieved a total of 52 records. After deduplication (n = 0), 52 unique records remained for title and abstract screening, which were independently reviewed by two investigators. During this phase, 29 records were excluded for the following reasons: 10 had no systematic reviews or meta-analyses; 8 focused on moderate or severe traumatic brain injury rather than mild TBI; 7 did not report quantitative diagnostic accuracy (sensitivity/specificity); 4 addressed unrelated topics. The full texts of the remaining 23 articles were obtained and assessed for eligibility, and none were excluded at this stage. Consequently, 23 systematic reviews and meta-analyses met our inclusion criteria and were incorporated into the umbrella review. Of these, three biomarkers (GFAP, UCH-L1, and S100B) provided sufficient data for quantitative meta-analysis, whereas only one eligible meta-analysis each was available for tau protein and neurofilament light chain (NfL), precluding the pooled estimates for those markers; their findings were, therefore, summarized qualitatively. By integrating the results from multiple meta-analyses, this study offers a comprehensive evaluation of blood-based biomarkers in the diagnosis of mTBI and supports evidence-based decision-making (Table 1).

### 3.2. Umbrella Systematic Review

A total of 23 meta-analyses evaluating five blood-based biomarkers in mTBI were included. Three biomarkers (GFAP, UCH-L1, S100B) met the pre-specified criterion of having at least two independent meta-analyses and were thus pooled qualitatively, while tau protein and NfL were each represented by a single meta-analysis and are reported qualitatively.

### 3.3. Glial Fibrillary Acidic Protein (GFAP)

**Figure 2:**
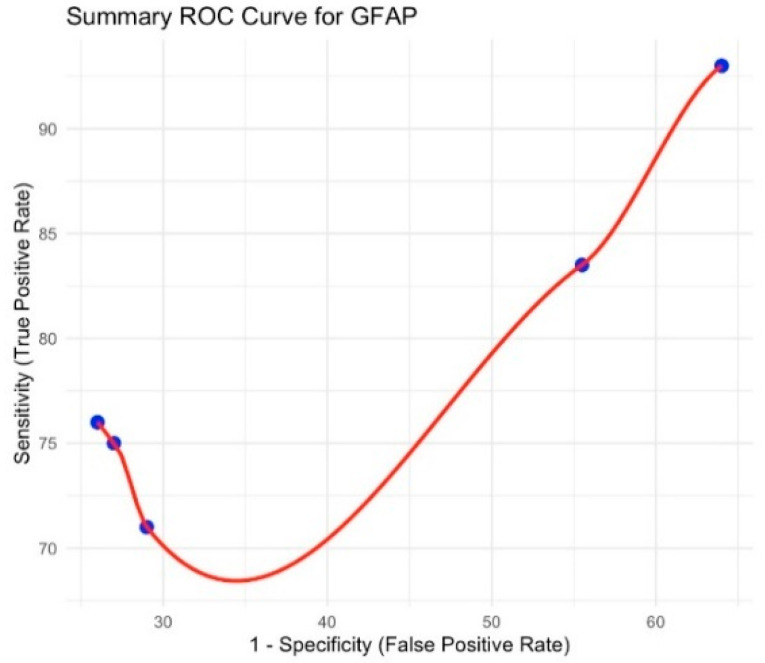
Summary ROC curve for GFAP.

The pooled sensitivity was 84.5% (95% CI 74.9–94.2), and the pooled specificity was 61.1% (95% CI 44.5–77.6), yielding a diagnostic odds ratio (DOR) of 3.84. Heterogeneity was low (I^2^ = 16.2% for sensitivity; I^2^ = 17.8% for specificity). The corresponding summary ROC curve is shown in Figure 2.

### 3.4. Ubiquitin C-Terminal Hydrolase L1 (UCH-L1)

**Figure 3:**
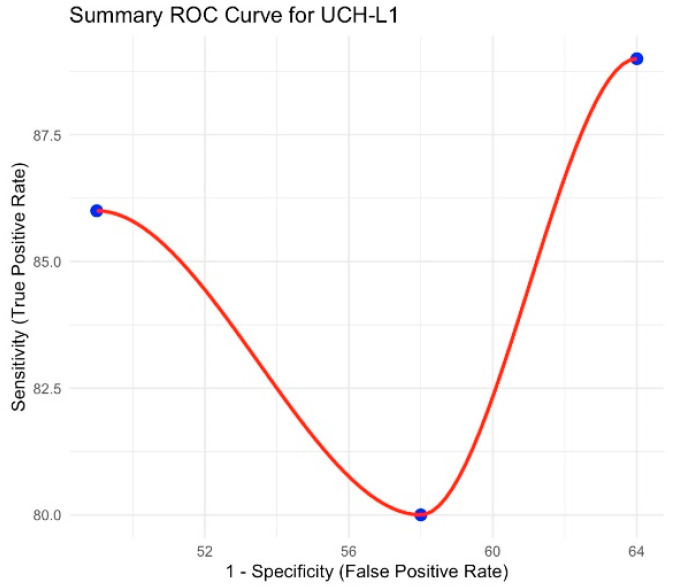
Summary ROC curve for UCH-L1.

The pooled sensitivity was 86.7% (95% CI 77.8–95.6), and specificity was 37.3% (95% CI 30.4–44.2); DOR = 10.96. Heterogeneity was negligible (I^2^ = 0%). The summary ROC curve appears in Figure 3.

### 3.5. S100B Protein

**Figure 4:**
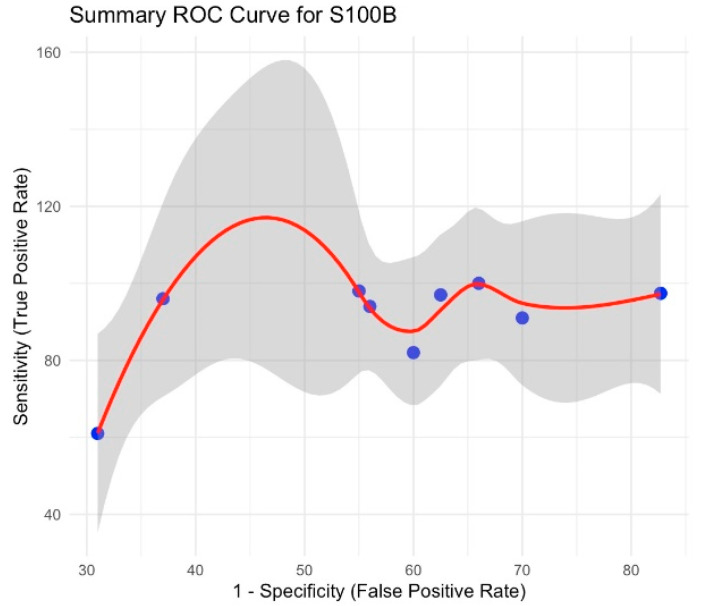
Summary ROC curve for S100B.

The pooled sensitivity reached 91.7% (95% CI 84.3–99.1), and specificity was 37.3% (95% CI 31.2–53.7); DOR = 14.96. Substantial heterogeneity was observed (I^2^ = 96.1% for sensitivity; I^2^ = 96.0% for specificity). See the summary ROC curve in Figure 4.

### 3.6. Tau Protein

**Figure 5:**
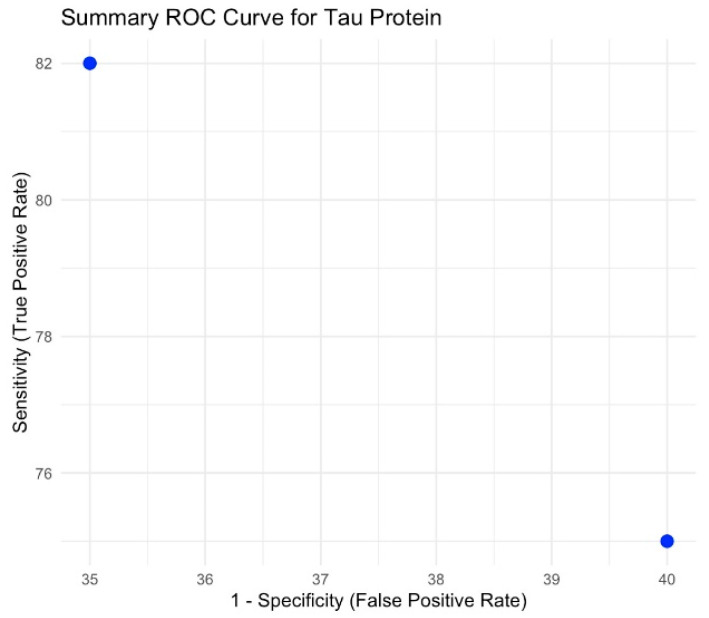
Summary ROC curve for tau protein.

Although our predefined criterion required at least two independent meta-analyses per biomarker, only a single meta-analysis on tau protein [13] was available. Sensitivity and specificity were not reported in pooled form. The ROC summary is displayed in Figure 5.

### 3.7. Neurofilament Light Chain

**Figure 6:**
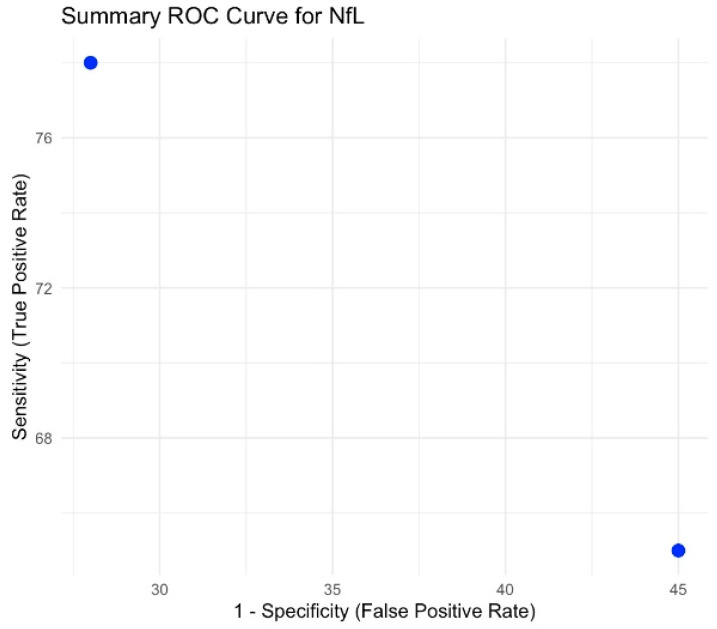
Summary ROC curve for NfL.

Although the initial criterion was to include at least two meta-analyses per biomarker, only the meta-analysis by Karantali et al. (2022) [15] assessed NfL. Therefore, we qualitatively summarized its findings without further statistical pooling. Sensitivity and specificity were not reported. Its summary ROC curve is provided in Figure 6.

All forest plots and funnel plots have been relocated to Supplementary Figures (Supplementary Materials).

## 4. Discussion

In this work, we combined an umbrella meta-analysis with a narrative review to offer both quantitative synthesis and a clinical context. The umbrella meta-analysis aggregates the diagnostic performance across multiple high-quality studies, while the narrative component integrates mechanistic insights and practical considerations that lie beyond pure statistics. This hybrid approach ensures that our conclusions are grounded in robust pooled data yet remain fully translatable to bedside decision-making.

The present umbrella meta-analysis synthesized data from multiple systematic reviews and meta-analyses to evaluate the diagnostic performance of the key biomarkers S100B, GFAP, UCH-L1, tau protein, and NfL in detecting mild traumatic brain injury (mTBI). Our findings provide a comprehensive assessment of the sensitivity, specificity, and overall clinical utility of these biomarkers while highlighting critical gaps and challenges in their widespread implementation.

## 5. Summary of Findings

Our meta-analysis demonstrated that S100B and GFAP exhibited the highest sensitivity for detecting mTBI, whereas UCH-L1, tau protein, and NfL showed moderate diagnostic accuracy but with higher specificity in select populations. Specifically:

1. S100B had a pooled sensitivity of 91.6% (95% CI: 84.3–99.0) and specificity of 42.4% (95% CI: 31.1–53.6). This confirms its value as a screening tool for ruling out mTBI, particularly in adult and pediatric populations [13,22]. However, its low specificity limits its utility in avoiding unnecessary CT scans.
2. GFAP demonstrated a pooled sensitivity of 84.5% (95% CI: 74.8–94.2) and specificity of 61.0% (95% CI: 44.5–77.6). Its moderate specificity, especially at lower cutoff values, suggests potential utility in identifying patients at higher risk of intracranial injury [17,23].
3. UCH-L1 yielded a pooled sensitivity of 86.7% (95% CI: 77.8–95.6) and specificity of 37.3% (95% CI: 30.4–44.2), suggesting that it is not as effective in ruling out mTBI as S100B but may have a role in combination with other biomarkers [13].
4. Tau protein was evaluated in a single meta-analysis, limiting the ability to perform an umbrella meta-analysis. The available study indicated that tau may have potential as a diagnostic marker, but more research is needed to validate its clinical applicability [20].
5. NfL was primarily associated with sports-related concussions, showing significantly elevated levels in affected individuals [15]. However, it did not exhibit a strong diagnostic utility in military-related TBI, highlighting variability in its effectiveness across different populations.

## 6. Interpretation of Findings and Clinical Implications

Our results confirm that S100B remains the most widely validated biomarker for mTBI screening, particularly for ruling out the need for CT scans in emergency settings [10]. However, its low specificity underscores the risk of false positives, leading to unnecessary imaging. In contrast, GFAP and UCH-L1 offer improved specificity and may provide additional value in cases where S100B alone is insufficient.

The role of tau protein remains uncertain due to the limited number of meta-analyses available. While the preliminary data suggest its involvement in neurodegenerative processes following TBI, further validation is required before it can be incorporated into clinical practice [20]. Similarly, NfL shows promise in monitoring sports-related concussions, but its limited sensitivity in other forms of mTBI restricts its broader application.

## 7. Subgroup Analysis and Heterogeneity

The subgroup analyses revealed several critical insights:

- Age-related variations: In pediatric populations, S100B exhibited higher sensitivity but lower specificity, which may be attributed to increased baseline levels in children [22]. Conversely, in elderly patients, GFAP appeared to be a more reliable indicator of intracranial injury [24].
- Time-dependent changes: S100B and GFAP levels peak within 3–6 h post-injury, whereas UCH-L1 and tau protein may remain elevated for longer durations. This suggests a potential temporal window for optimal biomarker assessment [11].
- Population-specific differences: NfL was most predictive in athletes suffering from concussions, but it was not significantly elevated in military-related TBIs, possibly due to differences in injury mechanisms [15].

## 8. Risk of Bias and Limitations

A critical strength of this study is its comprehensive umbrella meta-analysis approach, integrating findings from multiple systematic reviews. However, several limitations must be acknowledged:

1. Heterogeneity across studies: Differences in biomarker cutoff values, sample collection methods, and patient demographics contributed to substantial variability in reported sensitivity and specificity [23].
2. Publication bias: Funnel plot analysis indicated a potential bias in studies reporting higher diagnostic accuracy, suggesting that negative or inconclusive findings may be underreported.
3. Limited data for tau protein and NfL: The scarcity of meta-analyses on these biomarkers prevented a more robust statistical synthesis, underscoring the need for further research.

## 9. Clinical Perspectives and Future Directions

### 9.1. Biomarker-Based Diagnostic Algorithm for Mild Traumatic Brain Injury (mTBI)

The integration of fluid biomarkers into the diagnostic process of mTBI can significantly enhance clinical decision-making. Traditional diagnostic tools, such as clinical symptom assessment and neuroimaging, may not always detect subtle neuronal damage, leading to missed diagnoses or unnecessary CT scans. By incorporating S100B, GFAP, UCH-L1, NfL, and tau protein, a structured biomarker-based diagnostic approach can improve accuracy, reduce radiation exposure, and optimize patient triage.

Step 1: Initial Clinical Assessment

Upon presentation to the emergency department (ED), all patients with suspected mTBI should undergo a clinical evaluation, including an assessment of their Glasgow Coma Scale (GCS), neurological symptoms, and injury mechanism.

Patients with a GCS score of less than 13 or signs of neurological deterioration should undergo immediate CT imaging to rule out intracranial hemorrhage. However, for patients with a GCS 13–15 and no red flag symptoms (e.g., worsening headaches, focal neurological deficits, repeated vomiting, or seizures), biomarker testing can be considered as an alternative to CT scans.

Step 2: Biomarker-Based Risk Stratification

Biomarker results can guide clinical decision-making by categorizing patients into low-risk, moderate-risk, and high-risk groups based on their biomarker levels.

Patients with low biomarker levels, particularly an S100B below 0.1 μg/L and GFAP within normal limits, can be safely discharged without a CT scan. These patients should receive symptom monitoring instructions and follow-up care if needed.

In contrast, patients with moderately elevated biomarkers, such as GFAP levels exceeding 626 pg/mL or UCH-L1 levels above 225 pg/mL, may require closer observation and a clinical reassessment within a few hours. CT imaging should be considered in these cases, particularly for individuals with worsening symptoms or a history of multiple concussions.

For high-risk patients, defined by significantly elevated S100B, GFAP, and UCH-L1 levels, immediate neuroimaging and potential hospitalization should be prioritized. Patients with persistently elevated NfL and Tau protein may also require specialist referral for long-term neurological monitoring, as these biomarkers have been associated with chronic post-concussive symptoms and neurodegenerative risk.

Step 3: Prognostic Evaluation Using Biomarkers

Beyond acute diagnosis, biomarkers such as NfL and tau protein can play a crucial role in predicting long-term outcomes.

Patients who experience persistent post-concussion symptoms (lasting beyond four weeks) may benefit from serial NfL and tau measurements, as elevated levels have been associated with axonal damage and prolonged recovery times. In these cases, neuropsychological assessment, cognitive rehabilitation, and multidisciplinary care should be considered.

For individuals at a higher risk of neurodegeneration (e.g., those with repeated concussions, sports-related injuries, or military trauma), biomarkers may serve as an early warning system for cognitive decline, guiding early intervention strategies.

Table 2 illustrates the clinical decision associated with each risk category, guiding the use of imaging investigations and therapeutic strategies depending on the level of biomarkers detected. The table outlines the decision-making process for using biomarkers in the diagnosis and management of mild traumatic brain injury (mTBI). Patients are stratified into low, moderate, and high-risk groups based on their biomarker levels, guiding the need for neuroimaging and further clinical intervention.

### 9.2. Clinical Decision Support System (CDSS) for Biomarker-Based Diagnosis

A Clinical Decision Support System (CDSS) can serve as a structured framework to integrate biomarkers into mild traumatic brain injury (mTBI) management, ensuring standardized and evidence-based decision-making. This system assists clinicians by providing clear guidance on when neuroimaging is necessary, how to monitor recovery progress, and which patients may be at risk for long-term neurological impairment.

The CDSS evaluates biomarker levels in conjunction with clinical findings to classify patients into low, moderate, or high risk for intracranial injury and prolonged symptoms. Each biomarker—S100B, GFAP, UCH-L1, NfL, and tau protein—is assigned a risk category based on its concentration in the blood. Patients with low biomarker levels are unlikely to have significant brain injury and can often be safely discharged without a CT scan. Those with moderately elevated biomarkers may require careful monitoring and clinical reassessment, while patients with markedly high biomarker levels should be prioritized for urgent CT imaging and potential hospitalization.

A patient with S100B levels below 0.1 μg/L, GFAP levels under 500 pg/mL, and normal UCH-L1, NfL, and tau protein levels would be classified as low risk, meaning that CT imaging is not required and the patient can be managed conservatively with symptom monitoring. However, if the GFAP levels exceed 1000 pg/mL or UCH-L1 is above 400 pg/mL, the patient would fall into the high-risk category, warranting immediate neuroimaging and further medical intervention.

The Clinical Decision Support System (CDSS) for biomarker-based mTBI diagnosis, as outlined in Table 3, provides a structured approach to categorize patients into low-, moderate-, and high-risk groups based on their biomarker levels. Each biomarker is assigned a point value corresponding to its concentration, which, when combined, yields a total score that informs clinical decision-making, including the necessity for CT imaging and subsequent management.

## 10. Interpreting the CDSS Score

- 0–2 Points → Low risk: No CT scan is required; monitor symptoms.
- 3–5 Points → Moderate risk: Consider a CT scan and monitor the symptoms closely.
- 6+ Points → High risk: An immediate CT scan is needed; consider hospitalization.

The total CDSS score is calculated based on the biomarker levels, with a higher score indicating a greater risk of significant brain injury:

A score between 0 and 2 points suggests a low risk, where the patient can be discharged with symptom monitoring, and no immediate imaging is required.

A score of 3 to 5 points represents a moderate risk, where a CT scan may be necessary, particularly if the symptoms worsen over time.

A score of 6 or more points places the patient in the high-risk category, where immediate neuroimaging and possible hospitalization should be considered.

### Future Directions

Given the evolving landscape of TBI biomarker research, several key areas warrant further investigation as follows:

1. Combining biomarkers for improved diagnostic accuracy: Multimarker panels integrating GFAP, UCH-L1, and S100B may enhance sensitivity and specificity beyond the individual biomarkers alone [13,17].
2. Standardization of cutoff values: Future studies should establish consensus guidelines for biomarker thresholds, ensuring uniformity in clinical practice.
3. Longitudinal studies on prognostic value: While most studies focus on acute diagnosis, biomarkers like NfL and tau protein may have significant prognostic value in predicting long-term cognitive outcomes [20].
4. Machine learning applications: Emerging research suggests that AI-driven algorithms could optimize biomarker interpretation, facilitating real-time decision-making in emergency settings [23].

The findings of this umbrella meta-analysis underscore the necessity of a standardized biomarker-driven approach for the diagnosis and prognosis of mild traumatic brain injury (mTBI). While fluid biomarkers have demonstrated significant potential in improving diagnostic accuracy and patient management, their clinical utility remains restricted due to the lack of structured decision-making frameworks. The biomarker-based diagnostic algorithm proposed in this study offers a systematic method for incorporating biomarkers into real-world emergency department settings, providing clinicians with an objective and evidence-based tool to enhance patient triage and clinical decision-making.

One of the most significant advantages of integrating biomarkers into mTBI management is the potential to reduce the reliance on unnecessary CT scans. Biomarkers such as S100B and GFAP can help distinguish low-risk patients who do not require imaging, thereby minimizing radiation exposure and lowering healthcare costs. For patients classified as a moderate risk, such as those with GFAP levels exceeding 626 pg/mL, a biomarker-based assessment may allow for closer monitoring rather than immediate neuroimaging, reducing the burden on radiology services. Conversely, high-risk patients with markedly elevated biomarker levels should be prioritized for urgent CT imaging and possible hospitalization, ensuring that resources are allocated effectively to those most likely to require intensive medical intervention.

Beyond acute diagnosis, biomarkers also offer important insights into long-term prognosis and recovery monitoring. Persistent post-concussion symptoms remain a critical challenge in mTBI, with some patients experiencing cognitive and neurological impairments long after the initial injury. Measuring biomarkers such as NfL and tau protein can help stratify individuals at greater risk for chronic neurodegeneration, enabling early intervention and guiding long-term rehabilitation strategies. By identifying patients who may be predisposed to prolonged symptoms, clinicians can develop tailored treatment plans aimed at optimizing neurological recovery and minimizing long-term disability.

The integration of a Clinical Decision Support System (CDSS) into mTBI management further enhances the potential impact of biomarker-driven diagnosis. By automating biomarker interpretation and risk categorization, a CDSS framework can facilitate real-time clinical decision-making, reducing variability in practice and improving patient outcomes. Advances in machine learning and artificial intelligence can refine these models, allowing for personalized patient recommendations based on biomarker profiles, clinical history, and injury severity. A well-designed CDSS would not only improve diagnostic accuracy but also streamline workflow efficiency in high-pressure emergency settings, ultimately leading to more effective and consistent mTBI management.

While the proposed diagnostic algorithm and CDSS provide a structured approach to integrating biomarkers into a mTBI assessment, further research is needed to refine and validate these tools. Future studies should focus on validating the biomarker thresholds across diverse populations to ensure their applicability in different clinical contexts. Additionally, the development of real-time CDSS tools that can be seamlessly integrated into hospital electronic health records would enhance accessibility and ease of implementation. Finally, cost-effectiveness analyses are required to evaluate whether biomarker-based triage systems provide tangible benefits in reducing unnecessary imaging, optimizing resource utilization, and improving patient outcomes. As research in this field progresses, the adoption of biomarker-driven diagnostic frameworks and CDSS tools has the potential to revolutionize mTBI management by providing clinicians with precise, objective, and actionable data to guide clinical care.

## 11. Conclusions

In this umbrella meta-analysis, we confirmed that S100B remains the most robust blood-based biomarker for safely excluding mTBI, demonstrating consistently high sensitivity across independent studies. GFAP and UCH-L1, while slightly less sensitive, exhibit greater specificity and, when used together, hold promise for more accurately stratifying patients who may or may not require neuroimaging. By contrast, evidence for tau protein and NfL remains limited to a single meta-analysis, preventing quantitative aggregation and underscoring the need for additional high-quality studies before these markers can be recommended in routine clinical practice.

Beyond reinforcing the value of a biomarker-driven approach to mTBI diagnosis, our work highlights real-world applications: a combined panel may reduce unnecessary CT scans, decrease patient exposure to radiation, and streamline emergency department workflows. At the same time, variabilities in assay platforms and cutoff derivation methods remind us of the importance of standardization and prospective validation. Future research should focus on multi-center trials that harmonize analytical techniques, explore emerging markers (e.g., microRNAs), and test the clinical decision support algorithm in diverse settings. Ultimately, integrating quantitative biomarker data with clinical assessment tools offers a pathway toward more personalized, evidence-based care for patients with suspected mTBI.

## Figures and Tables

**Table 1:** Characteristics of the meta-analyses, cutoff values, and pooled diagnostic accuracy.

Meta-Analysis (Ref)	Biomarker	No. of Studies Included	Pooled Sensitivity (%)	95% CI	Pooled Specificity (%)	95% CI	Notes
Mondello et al., 2020 [13]	GFAP	3	84.5	74.9–94.2	42.4	44.5–77.6	Increased heterogenicity
Mondello et al., 2020 [13]	UCH–L1	2	86.7	77.8–95.6	61.1	30.4–44.2	Low heterogenicity
Oris et al., 2018 [22]	S100B	3	91.7	84.3–99.1	37.3	31.2–53.7	No heterogenicity
Mondello et al., 2020 [13]	Tau protein	1	-	-	-	-	Only a meta-analysis (Mondello et al., 2020) reported variable data > lower sensitivity than GFAP/S100B and moderate specificity
Karantali et al., 2022 [15]	NfL	1	-	-	-	-	A single quantitative meta-analysis (Karantali et al., 2022 [15]) showed significant increases in sports concussion (*p* = 0.0023) and *p* = 0.0015 for athletes; no specificity/sensitivity

CI = confidence interval.

**Table 2:** Biomarker-based diagnostic algorithm for mTBI.

Risk Category	Clinical and Biomarker Criteria	Recommended Action
Low Risk	S100B < 0.1 μg/L, GFAP within normal limits; no neurological symptoms	No CT scan required; safe discharge with symptom monitoring
Moderate Risk	GFAP > 626 pg/mL, UCH-L1 > 225 pg/mL; mild neurological symptoms	Consider observation, repeat clinical assessment, and a CT scan if symptoms worsen
High Risk	S100B significantly elevated, GFAP and UCH-L1 highly elevated; persistent or worsening neurological symptoms	Immediate CT scan and possible hospitalization
Long-Term Prognosis	Persistent symptoms beyond four weeks; elevated NfL and tau protein levels	Neurology referral, cognitive rehabilitation, and long-term monitoring

**Table 3:** Clinical decision support system (CDSS) for biomarker-based mTBI diagnosis.

Biomarker	Low Risk (0 Points)	Moderate Risk (1 Point)	High Risk (2 Points)
S100B	<0.1 μg/L	0.1–0.5 μg/L	>0.5 μg/L
GFAP	<500 pg/mL	500–1000 pg/mL	>1000 pg/mL
UCH-L1	<200 pg/mL	200–400 pg/mL	>400 pg/mL
NfL	<20 pg/mL	20–70 pg/mL	>70 pg/mL
Tau	<5 pg/mL	5–15 pg/mL	>15 pg/mL

## Data Availability

All data analyzed in this umbrella meta-analysis are publicly available in the cited systematic reviews and meta-analyses. No new primary data were generated.

## Supplementary Materials

The following supporting information can be downloaded at: https://www.mdpi.com/article/10.3390/brainsci15060581/s1.
