# Supplementary material for: The Diagnostic and Prognostic Role of Biomarkers in Mild Traumatic Brain Injury: An Umbrella Meta-Analysis
Source: Brain Sci. 2025 May 28;15(6):581. doi: 10.3390/brainsci15060581 (PMC12190496; doi:10.3390/brainsci15060581)
Supplement: Supplementary file 1 [file brainsci-15-00581-s001.zip › brainsci-3612623-supplementary.pdf]

Supplementary materials:

Figure S1: Forest Plot of GFAP Sensitivity

Pooled sensitivity of GFAP biomarker in detecting mTBI.

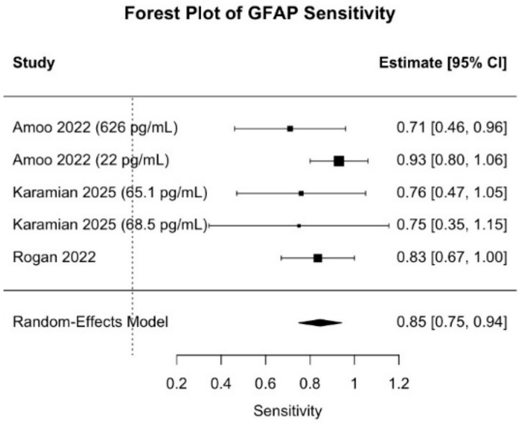

The forest plot for GFAP sensitivity indicates a moderate performance in detecting mTBI, suggesting that this biomarker effectively identifies patients with intracranial injuries, thus contributing to the initial diagnosis.

Figure S2: Forest Plot of GFAP Specificity

Pooled specificity of GFAP biomarker in detecting mTBI.

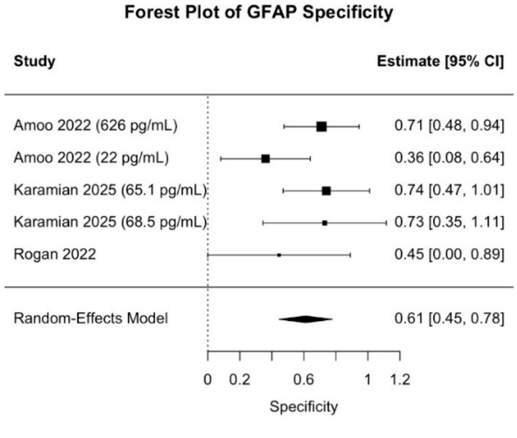

The forest plot of GFAP specificity shows more balanced value compared to S100B. Thus, GFAP may provide an optimal compromise between correct case identification and avoidance of diagnostic errors, opting for the adopting threshold.

Figure S3: Funnel Plot for GFAP Sensitivity

Assessment of publication bias in studies reporting GFAP sensitivity.

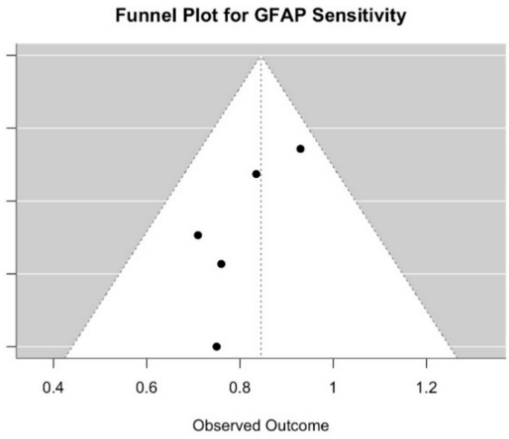

The funnel plot for GFAP sensitivity is used to detect possible publication bias. A symmetrical distribution of the data would strengthen the methodological validity of the included studies.

Figure S4: Funnel Plot for GFAP Specificity

Assessment of publication bias in studies reporting GFAP specificity.

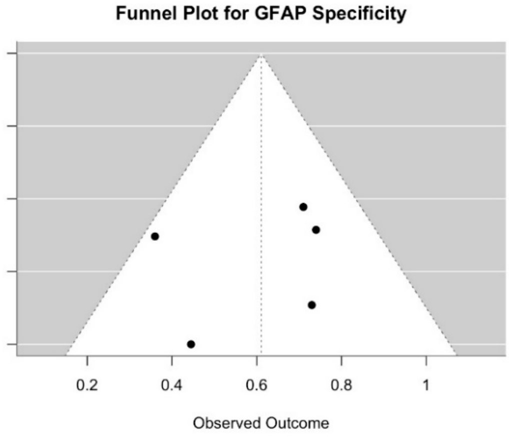

This graph analyzes the distribution of GFAP specificity data. A symmetry of the reported points would support the reliability of the results, and possible asymmetries might suggest the need for further investigation.

Figure S5: Forest Plot of UCH-L1 Sensitivity

Pooled sensitivity of UCH-L1 biomarker in detecting mTBI.

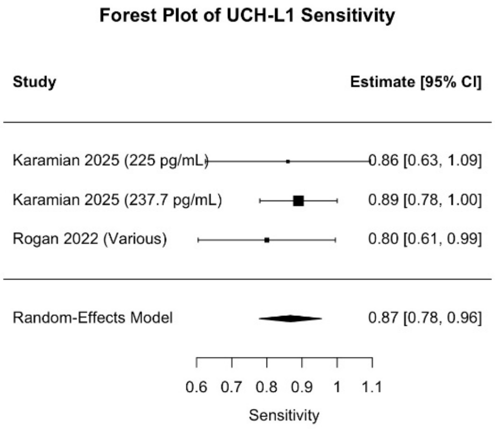

The forest plot shows the cumulative sensitivity of UCH-L1 in detecting mTBI, highlighting a good performance that supports the use of this biomarker in early identification of injuries.

Figure S6: Forest Plot of UCH-L1 Specificity

Pooled specificity of UCH-L1 biomarker in detecting mTBI.

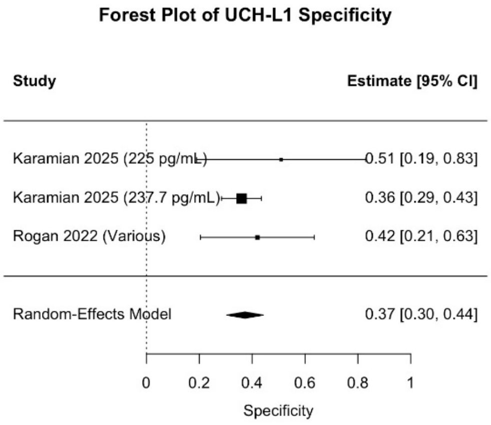

The forest plot for UCH-L1 specificity shows low values, suggesting that this marker, on its own, may have limitations in confirming an accurate diagnosis. Therefore, its integrating into a biomarker panel may be a more appropriate approach.

Figure S7: Funnel Plot for UCH-L1 Sensitivity

Assessment of publication bias in studies reporting UCH-L1 sensitivity.

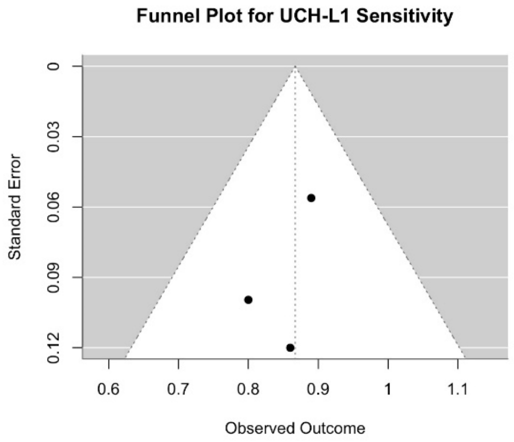

The funnel plot for UCH-L1 sensitivity was used to assess the risk of bias in the literature. A uniform distribution of the data would strengthen the confidence in the reported estimates.

Figure S8: Funnel Plot for UCH-L1 Specificity

Assessment of publication bias in studies reporting UCH-L1 specificity.

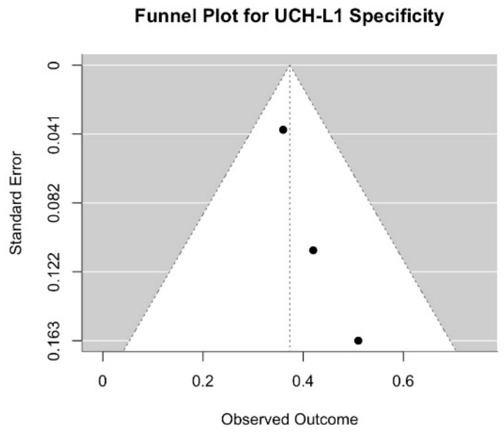

This funnel plot analyzes the distribution of UCH-L1 specificity data, checking for possible methodological variations or publication errors that could influence the values obtained.

Figure S9: Forest Plot of S100B Sensitivity

Pooled sensitivity of S100B biomarker in detecting mild traumatic brain injury (mTBI).

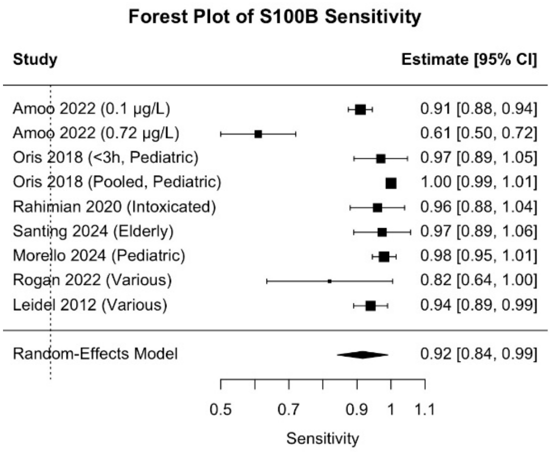

The forest plot summarizes the cumulative sensitivity of the S100B biomarker in detecting mTBI. The results indicate a remarkably high sensitivity, thus highlighting its essential role as a screening tool to exclude intracranial lesions.

Figure S10: Forest Plot of S100B Specificity

Pooled specificity of S100B biomarker in detecting mTBI.

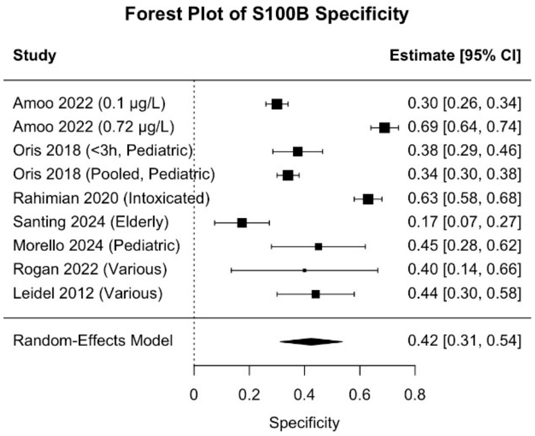

The forest plot for S100B specificity reveals a low level, suggesting that although the marker is excellent in detecting positive cases, there is an increased risk of false positive results. This aspect limits the utility of S100B in establishing a definitive diagnosis.

Figure S11: Funnel Plot for S100B Sensitivity

Assessment of publication bias in studies reporting S100B sensitivity.

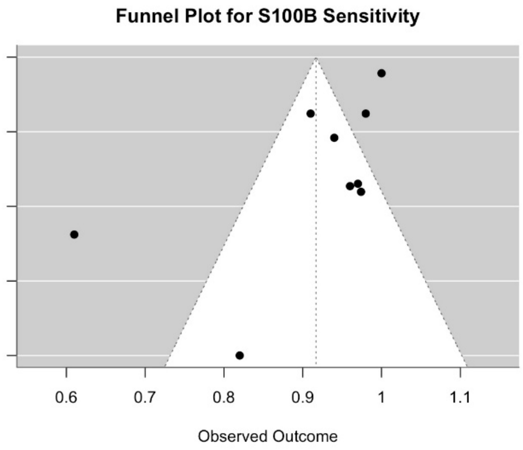

The funnel plot for S100B sensitivity was used to assess the presence of publication bias in the reviewed studies. The distribution of the data, if symmetric, indicates a methodological robustness of the reported estimates.

Figure S12: Funnel Plot for S100B Specificity

Assessment of publication bias in studies reporting S100B specificity.

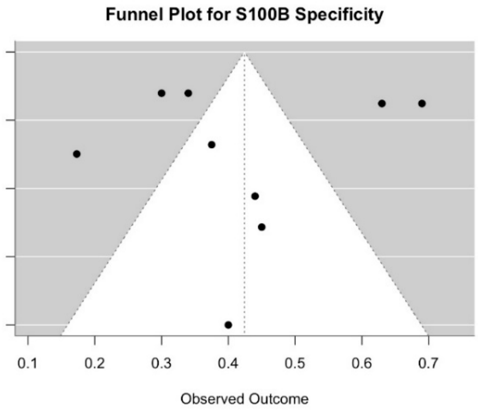

This funnel plot examines the consistency of the data regarding S100B specificity. A symmetric distribution would strengthen confidence in the results obtained, while possible asymmetries could suggest methodological biases that require investigation.

Figure S13: Forest Plot of Tau Protein Sensitivity

Pooled sensitivity of tau protein biomarker in detecting mTBI.

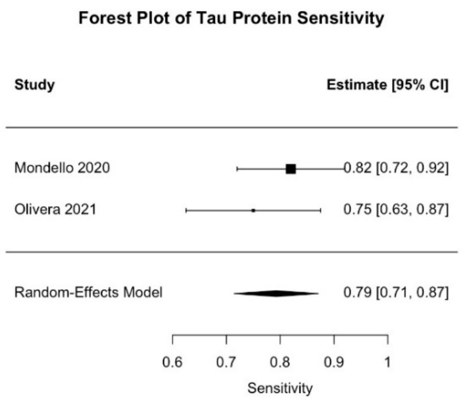

The forest plot for Tau protein sensitivity indicates moderate results, based on the available studies. These preliminary data suggest a potential diagnostic, but further validation by future studies is needed.

Figure S14: Forest Plot of Tau Protein Specificity

Pooled specificity of tau protein biomarker in detecting mTBI.

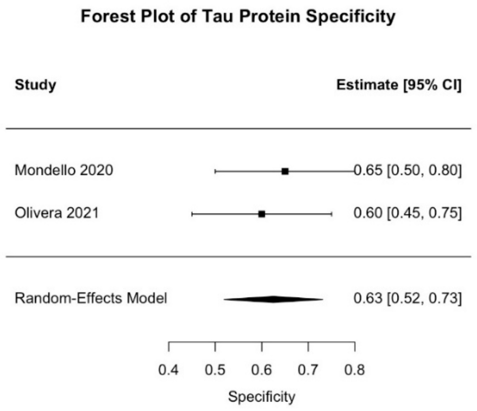

The forest plot for Tau protein specificity reveals variability in the reported results, highlighting the current uncertainty regarding the applicability of this biomarker in clinical practice.

Figure S15: Funnel Plot for Tau Protein Sensitivity

Assessment of publication bias in studies reporting tau protein sensitivity.

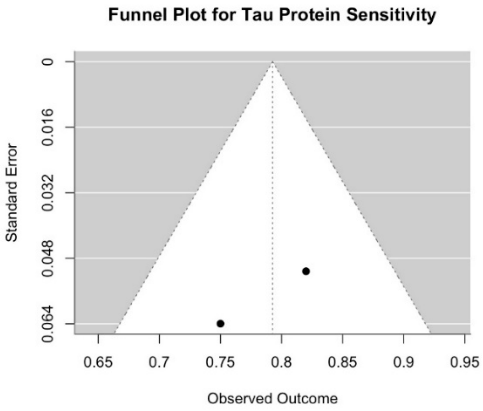

The funnel plot for Tau protein sensitivity assesses the possible influence of publication bias. The limitations of the available data require caution in interpreting the obtained results

Figure S16: Funnel Plot for Tau Protein Specificity

Assessment of publication bias in studies reporting tau protein specificity.

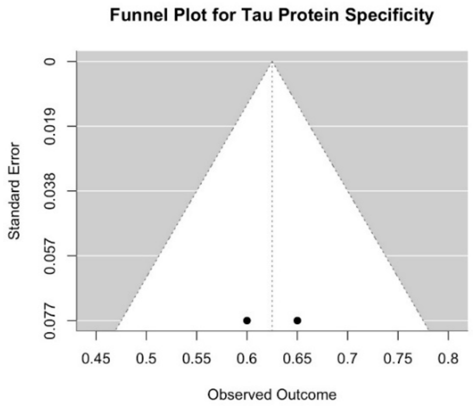

This funnel plot examines the distribution of the data for Tau protein specificity, highlighting the need for further studies to confirm the reported values.

Figure S17: Forest Plot of NfL Sensitivity

Pooled sensitivity of Neurofilament Light Chain (NfL) biomarker in detecting mTBI.

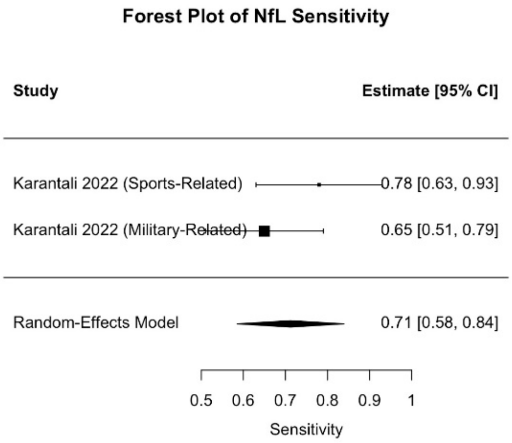

The forest plot for NfL sensitivity highlights the ability of this biomarker to detect mTBI, especially in specific subgroups, such as athletes with concussions. This highlights its relevance in particular context.

Figure S18: Forest Plot of NfL Specificity

Pooled specificity of Neurofilament Light Chain (NfL) biomarker in detecting mTBI.

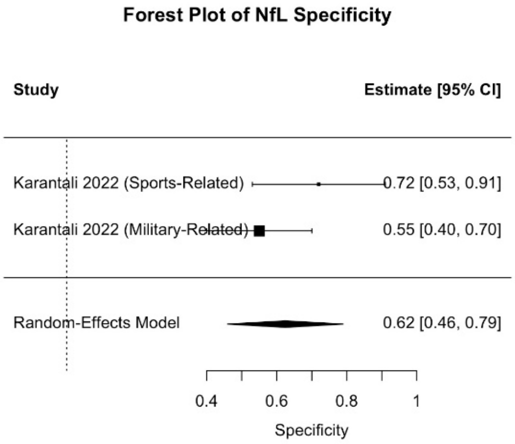

The forest plot for NfL specificity shows variability across population and methodology, highlighting the influence of collection and timing factors on reported values.

Figure S19: Funnel Plot for NfL Sensitivity

Assessment of publication bias in studies reporting NfL sensitivity.

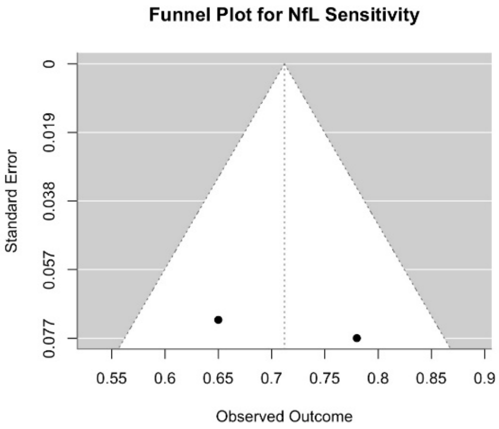

The funnel plot for NfL sensitivity is used to identify potential publication bias. A symmetrical distribution of the data would strengthen confidence in the estimates presented.

Figure S20: Funnel Plot for NfL Specificity

Assessment of publication bias in studies reporting NfL specificity.

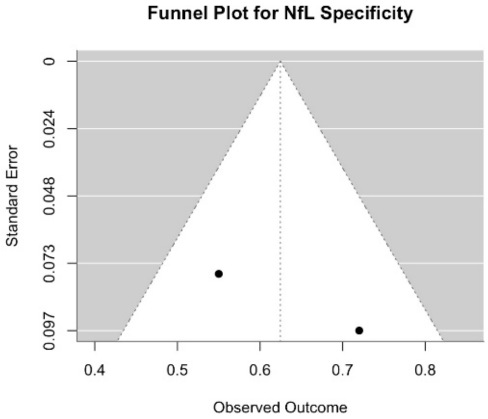

This funnel plot analyzes the consistency of the NfL specificity data, highlighting whether any asymmetries could affect the interpretation of the results and highlighting the need for methodological standardization.
